# Nutraceuticals in the Prevention and Treatment of the Muscle Atrophy

**DOI:** 10.3390/nu13061914

**Published:** 2021-06-02

**Authors:** Yanan Wang, Qing Liu, Helong Quan, Seong-Gook Kang, Kunlun Huang, Tao Tong

**Affiliations:** 1 Beijing Advanced Innovation Center for Food Nutrition and Human Health, College of Food Science and Nutritional Engineering, China Agricultural University, Beijing 100083, China; wangyanan_ytu@126.com; 2 Jilin Green Food Engineering Research Institute, Changchun 130022, China; jl_greenfood@163.com; 3 Exercise and Metabolism Research Center, College of Physical Education and Health Sciences, Zhejiang Normal University, 688 Yingbin Avenue, Jinhua 321004, China; quanhelong@zjnu.edu.cn; 4 Department of Food Engineering, Mokpo National University, Muangun 58554, Korea; sgkang@mokpo.ac.kr; 5 Key Laboratory of Safety Assessment of Genetically Modified Organism (Food Safety), Ministry of Agriculture, Beijing 100083, China

**Keywords:** muscle atrophy, protein synthesis/degradation, molecular mechanisms, nutraceutical, phytochemical

## Abstract

Imbalance of protein homeostasis, with excessive protein degradation compared with protein synthesis, leads to the development of muscle atrophy resulting in a decrease in muscle mass and consequent muscle weakness and disability. Potential triggers of muscle atrophy include inflammation, malnutrition, aging, cancer, and an unhealthy lifestyle such as sedentariness and high fat diet. Nutraceuticals with preventive and therapeutic effects against muscle atrophy have recently received increasing attention since they are potentially more suitable for long-term use. The implementation of nutraceutical intervention might aid in the development and design of precision medicine strategies to reduce the burden of muscle atrophy. In this review, we will summarize the current knowledge on the importance of nutraceuticals in the prevention of skeletal muscle mass loss and recovery of muscle function. We also highlight the cellular and molecular mechanisms of these nutraceuticals and their possible pharmacological use, which is of great importance for the prevention and treatment of muscle atrophy.

## 1. Introduction

Muscle atrophy is a worldwide chronic progressive muscle disease due to excessive protein degradation versus protein synthesis. It is characterized by muscle mass loss, muscle weakness, and disability, all of which are associated with an impaired quality of life [1]. Various stimuli, such as aging, genetics, denervation, starvation, unhealthy lifestyle (i.e., sedentariness, high fat diet (HFD)), systemic pathologies (i.e., diabetes, inflammation, cancer), and long-term drug therapy (i.e., glucocorticoids) are known to further skeletal muscle loss and weakness [2,3,4]. On one hand, the increase in life expectancy and the elderly population, in conjunction with the high prevalence of muscle atrophy in the elderly, create an enormous socioeconomic burden. On the other hand, despite its broad clinical impact, skeletal muscle atrophy is a relatively understudied and underdeveloped area of biomedical research and still lacks effective pharmacological therapy, which pointing to a large need for effective and new anti-atrophy approaches.

**Figure 1:**
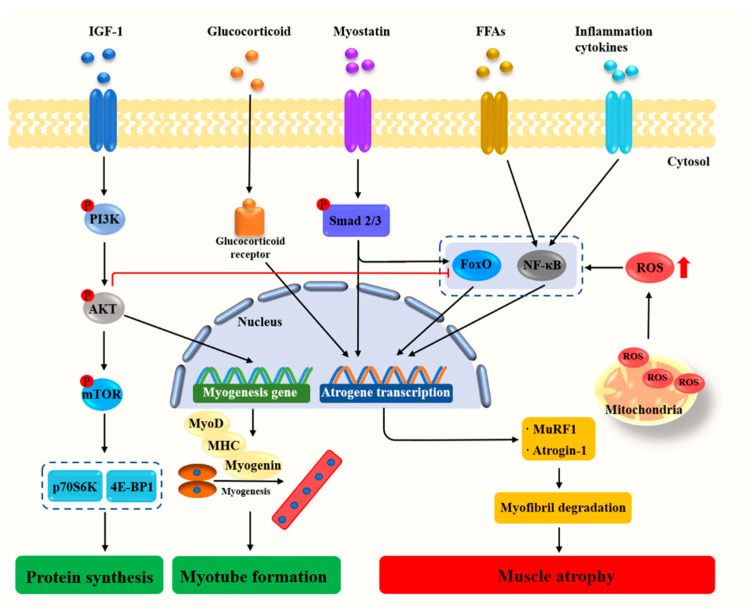
Cellular and molecular mechanisms regulating muscle growth and atrophy. Akt, Protein kinase B; FFAs, Free fatty acids; FoxO, Forkhead box O; IGF-1, Insulin-like growth factor 1; MAFbx, Muscle atrophy F-box; MHC, Myosin heavy chain; mTOR, Mammalian target of rapamycin; MuRF1, Muscle RING-finger protein-1; MyoD, Myogenic differentiation antigen; NF-κB, Nuclear factor kappa-B; PI3K, Phosphoinositide 3-kinase; P70S6K, Ribosomal protein S6 kinase; ROS, Reactive oxygen species; 4E-BP1, Rapamycin complex and 4E binding protein-1.

Molecular investigations of skeletal muscle atrophy have primarily focused on the atrogin-1/muscle atrophy F-box (MAFbx) and E3 ubiquitin ligases muscle RING finger 1 (MuRF1) [5]. MuRF1 binds to myosin heavy chain (MHC) and myofibrillar protein titin, resulting in skeletal muscle loss [6]. Mice deficient in either MAFbx or MuRF1 were demonstrated to be resistant to atrophy [7]. Under a wide range of conditions including starvation, muscle disuse, glucocorticoid excess, and aging, the glucocorticoid receptor and transcription factor forkhead box O (FoxO) increase the expression of MuRF1 and promote muscle atrophy [5]. Moreover, proinflammatory cytokines present in the muscle trigger the nuclear factor κ-light-chain-enhancer of activated B cells (NF-κB) signaling pathways to mediate muscle degradation [8,9]. Oxidative stress, characterized by increased reactive oxygen species (ROS) production and impairment of antioxidant defense systems, is also a major trigger of imbalance between protein synthesis and degradation leading to muscle atrophy [10,11]. Myostatin, a member of the transforming growth factor-*β* (TGF-*β*) superfamily and critical autocrine inhibitor of skeletal muscle growth, induces muscle fiber atrophy by the activation of Smad2 and Smad3 and suppresses protein synthesis by the inhibition of protein kinase B (Akt) (Figure 1). On the contrary, the activation of insulin-like growth factor (IGF-1)/phosphoinositide 3-kinase (PI3K)/Akt signaling pathway represses the MAFbx and MuRF1 gene expression via decreasing the expression and activity of FoxO. In addition to inhibiting muscle atrophy, the activation of insulin-like growth factor 1 (IGF-1)/PI3K/Akt pathway also stimulates protein synthesis and induces muscle growth via rapamycin complex (mTOR) kinase [5]. Recently, gut microbiota has been proposed as an environmental factor involved in the regulation of host immunity and muscle metabolism [12,13]. Understanding the change in the signaling pathways during the development of muscle atrophy may lead to identifying and developing therapeutic agents for muscle atrophy.

Experimental models used for muscle atrophy studies mainly contain HFD-, cancer-, denervation-, glucocorticoid-, and disuse-induced muscle atrophy model. The mechanisms inducing muscle atrophy between the numerous models presented are not identical. For example, the mechanisms underlying HFD-induced atrophy are not fully understood, but recent experimental data suggest that multiple factors such as elevated levels of toxic lipid metabolites and pro-inflammatory cytokines may contribute to a decrease in muscle mass and strength [14]. The pro-inflammatory cytokines, such as tumor necrosis factor-*α* (TNF-*α*), interleukine-6 (IL-6), and interleukine-1 (IL-1), have long been considered as mediators of cancer-induced muscle atrophy [1]. Enhanced generation of mitochondrial ROS may be a common factor in the mechanism underlying denervation-induced atrophy [15]. Although the exact mechanism underlying glucocorticoid-induced skeletal muscle atrophy is unclear, it is considered to be multifactorial and is related to decreased protein synthesis, increased protein degradation, alterations in carbohydrate metabolism, oxidative stress, and/or decreased sarcolemmal excitability [16].

Evidence from various preclinical studies has revealed that nutraceuticals play a significant role in maintaining muscle health and emerge as potential therapeutic agents in a broad range of muscle atrophy models [17,18]. For example, polyphenols, flavonoids, alkaloids, and probiotics show beneficial regulatory effects on muscle cells or tissues and they seem to be potentially more suitable for long-term use than traditional therapeutic agents. Here, this review highlights current knowledge and recent advances in understanding the impact of various nutraceuticals on skeletal muscle atrophy. We also will discuss their underlying molecular mechanisms in the prevention of muscle mass loss and recovery of muscle function.

## 2. Materials and Methods

Research articles that focused on the nutraceutical intervention for muscle atrophy and the mechanisms of muscle atrophy were collected using various search engines including PubMed, Web of Science, Scopus, Google Scholar, Springer, and Wiley. The keywords such as muscle atrophy, protein synthesis/degradation, molecular mechanisms, nutraceutical, nutrient, oxidative stress, muscle mass, muscle strength, microbiota, glucocorticoid, and phytochemical were used. The articles identified were reviewed and relevant citations within these articles were also reviewed.

## 3. Dietary Nutrients

Currently, there is increasing preclinical data and evidence that dietary nutrients supplementation can protect skeletal muscle from atrophy damage. In this part, we will summarize the existing scientific evidence gleaned from a variety of in vitro/vivo studies that supports the beneficial impacts of dietary nutrients on the skeletal muscle atrophy.

### 3.1. Protein, Amino Acid, and Peptides

The most obvious feature of skeletal muscle atrophy is the loss of muscle protein due to the imbalance between decomposition of muscle protein and the synthesis of muscle protein. Protein, amino acid, and peptides supplementation (Table 1) is considered to be effective in increasing muscle anabolism and preventing muscle atrophy during extended periods of immobilization [19]. Oral administration of a high protein liquid nutritional supplement (milk protein, whey protein, leucine, and citrulline) inhibits intermittent loading-induced muscle atrophy and increases muscle mass by activating the mTORC1 signaling pathway (Ribosomal protein S6 kinase (p70S6K) and ribosomal protein S6 (rpS6)) [20]. Shin et al. reported that soluble whey protein hydrolysate increases grip strength, muscle mass, and cross-sectional area of muscle fiber and meliorates immobilization-induced C57BL/6 mice muscle atrophy via regulating the PI3K/Akt pathway [21]. Leucine promotes muscle protein synthesis in C2C12 myotubes through the activation of mTOR and rapamycin complex and 4E binding protein-1 (4E-BP1) signaling pathway, a critical intracellular pathway involved in the regulation of muscle protein synthesis [22]. Meanwhile, Dreyer et al. found that following resistance exercise leucine-enriched essential amino acid and carbohydrate ingestion enhances mTOR signaling and protein synthesis in human leg muscle [23]. In addition, supplementation of leucine can offset anabolic resistance and increases muscle protein synthesis in old women [24]. Combined resistance training with essential amino acid mixture (L-isoleucine, L-histidine, L-leucine, L-lysine, L-methionine, L-phenylalanine, L-threonine, and L-valine) supplementation protects against the losses of muscle mass and strength in man during 28 days of bed rest and energy deficit [25]. In cachexia-inducing tumor Naval Medical Research Institute mice with a significant suppression in the loss of body weight, leucine or valine could increase protein synthesis and attenuate the increased protein degradation [26]. Similar to leucine and valine, isoleucine acts as an intermediate to drive muscle protein synthesis and reduce the rate of protein breakdown [27]. Branched-chain amino acids (22.9% L-isoleucine, 45.8% L-leucine, and 27.6% L-valine) have been shown to inhibit hindlimb suspension-induced muscle atrophy in Sprague Dawley (SD) rats via, at least in part, suppressing the expression of MAFbx and MuRF1 in soleus muscles [28]. The supplementation of amino acid complex (leucine: isoleucine: valine = 2:1:1; 3% in tap water) ameliorates angiotensin II-induced muscle atrophy and increases skeletal muscle weight and gastrocnemius tissue cross-sectional area [29]. Similarly, a study showed that before the inactive phase of rat micellar casein and whey protein (1:1) supplementation increase muscle protein synthesis due to increased plasma levels of branched-chain amino acids and the activation of the downstream targets of mTOR signaling [30].

D-methionine, an amino acid commonly found in fermented dairy products like cheese and yogurt, increases gastrocnemius muscle and soleus muscle weight in cisplatin-induced Wistar rats muscle atrophy model. The protective effect of D-methionine is associated with the downregulation of the expressions of MAFbx and MuRF1 and the upregulation of the expressions of myogenin and myogenic differentiation antigen (MyoD) [31]. In addition, dietary supplement with high level methionine (1.25%) significantly increases the muscle total cross-sectional area of the juvenile rainbow trout (*Oncorhynchus mykiss*) [32]. In the broilers the high level of methionine diets increases breast muscle growth by increasing the mRNA levels of myogenic regulatory factors such as myogenic regulatory factors 4 and myogenic factors 5 and decreasing myostatin mRNA level [33].

Glutamine is an important amino acid with many functions in the body, such as stimulating protein-synthetic and inhibiting protein-degradative signaling pathways in skeletal muscle of diabetic rats [34]. l-glutamine improves C2C12 cells differentiation and prevents TNF-*α*-induced myotube atrophy via reducing p38 mitogen-activated protein kinase (MAPK) signal transduction [35]. Oral l-glutamine pretreatment attenuates 24-h fasting-induced muscle atrophy in C57BL/6 mice [36]. Moreover, glutamine supplementation prevents glucocorticoid-induced SD rats skeletal muscle atrophy and increases muscle mass, partially through inhibiting the expression of myostatin. Similarly, in C2C12 myoblast cells, glutamine treatment prevents the hyperexpression of myostatin induced by dexamethasone [37].

Taurine, a containing sulfur nonessential amino acid, is widely distributed in different tissues in particular in skeletal muscle and prevents muscle atrophy in vitro and in vivo. In C2C12 myotubes, Stacchiotti et al. reported that taurine rescues cisplatin-induced muscle atrophy by restoring microtubular and mitochondrial function and reducing the overload and the localization of autophagolysosomes [38]. Intraperitoneal administration of taurine to albino mice every day for 1 week alleviates muscle atrophy induced by reduced mechanical loading through downregulating MuRF1 and caspase 3 expression in quadriceps muscle [39]. Furthermore, oral supplementation of taurine attenuates diquat-induced muscle damage and suppresses the gene expression of muscle atrophy-related genes (MAFbx and MuRF1) in the skeletal muscle of piglets [40].

Dietary supplementation of creatine in tumor-bearing Wistar rats prevents muscle atrophy via alleviating inflammation and proteolysis as evidenced by reduced inflammation factor (TNF-*α* and IL-6) and muscle atrophy gene (MAFbx and MuRF1) [41]. In a separate study, Marzuca-Nassr et al. reported that oral gavage of creatine to Wistar rats increases the muscle mass and the protein level of 4E-BP1 in extensor digitorum longus and prevents hindlimb suspension-induced muscle atrophy [42].

S-allyl cysteine (SAC) is an organosulfur compound of *Allium sativum*, possessing immuno-and redox-modulatory broad-spectrum properties. Gupta et al. reported that SAC suppresses the rise in cytokines levels (TNF-*α*, IL-6, and myostatin) and protects myotubes from H_2_O_2_-induced protein loss in C2C12 myotubes. They also demonstrated that SAC alleviates mass loss and increases cross-sectional area of muscle on Swiss albino mice [43]. Those results illustrate that SAC significantly inhibits the proteolytic systems and inflammatory/oxidative molecules and therefore play a crucial role in the prevention of skeletal muscle atrophy.

In addition to protein and amino acids, diet contain multiple bioactive peptides have been shown to prevent muscle atrophy. For example, dietary supplementation of small molecular weight soybean protein-derived peptides attenuates burn injury-induced muscle atrophy in Wistar rats by modulating ubiquitin–proteasome system and autophagy signaling pathway [44]. PYP1-5, a *Pyropia yezoensis* peptide, inhibits muscle atrophy induced by dexamethasone through the downregulation of MAFbx and MuRF1 in C2C12 myotubes [45]. There are also some experimental muscular atrophy-related therapies based on the use mimetic peptides. For example, N-myristoylated Cblin, a peptide mimetic of tyrosine, inhibits dexamethasone-induced atrophy in C2C12 myotubes and increases gastrocnemius muscle wet weight and cross-sectional area in dexamethasone-treated C57BL/6 mice. The primary mechanism is associated with the downregulated expression of MAFbx and MuRF1 [46]. Laminin mimetic peptide nanofibers significantly promote the activation of satellite cell in skeletal muscle and accelerate the regeneration myofibrillar following acute muscle injury in SD rats [47].

Succinate, a vital intermediate in the tricarboxylic acid cycle, has been reported to play an important role in the process of muscle. Succinate supplementation interferes myoblast differentiation, characterized by significant decreases in the later markers of myogenesis and fewer nuclei per myosin heavy chain positive structure in C2C12 cells. Furthermore, recent studies demonstrated that orally administration of 2% succinate impairs muscle regeneration in FVB mice [48].

**Table 1:** Protein, amino acids, and peptides in the prevention of the muscle atrophy in vitro/vivo.

Protein/Amino Acids/Peptides	Structure	Example Source	Inducer	Model	Effect	Mechanism	Ref
Whey protein	/	Egg, meat, milk	Immobilization	C57BL/6 mice	↑ Grip strength ↑ Muscle mass ↑ Cross-sectional area of muscle fiber	↑ mTOR signaling	[21]
Leucine	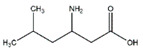	Meat, milk	/	Human	↑ Muscle protein	↑ Protein synthesis ↑ mTOR signaling	[23]
				C2C12 cells	↑ Muscle protein	↑ Protein synthesis ↑ mTOR signaling	[22]
Valine	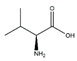	Meat, milk	Cachexia	NMRI mice	↑ Muscle weight	↑ Protein synthesis ↓ Protein degradation	[26]
Isoleucine	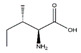	Meat, milk	/	SD rats	↑ Muscle weight	↑ Protein synthesis ↓ Protein degradation	[30]
Branched-chain amino acids	/	Meat, milk	Hindlimb suspension	SD rats	↑ Muscle weight	↑ mTOR signaling ↓ MuRF1, MAFbx	[28,30]
D-methionine	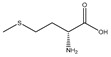	Soybean	Cisplatin	Wistar rats	↑ Muscle weight	↑ MyoD ↓ MuRF1, MAFbx	[31]
Methionine	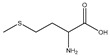	Soybean	/	Broilers	↑ Breast muscle growth	↑ Myf5, MEF2A	[33]
Glutamine	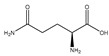	Meat	TNF-*α*	C2C12	↑ Cell differentiation	↓ p38 MAPK singnaling	[35]
			Fasting	C57BL/6 mice	↑ Muscle weight	↑ mTOR signaling	[36]
Taurine	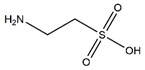	Fish, shellfish	Reduced mechanical loading	Albino mice	↓ Muscle injury	↓ MuRF1, caspase 3, MAFbx	[39]
			Diquat	Piglets	↑ Muscle protein	↓ MuRF1, MAFbx	[40]
			Cisplatin	C2C12 cells	↑ Myotubes size	↑ Microtubular, mitochondrial function	[38]
S-allyl cysteine	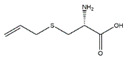	*Allium sativum*	Denervation	Swiss albino mice	↑ Muscle mass	↓ MuRF1, MAFbx	[43]
			H_2_O_2_	C2C12 cells	↑ Myotubes size	↓ Oxidative stress, inflammation ↓ MuRF1, MAFbx	[43]
Soybean protein-derived peptides	/	Soybean	Burn injury	Wistar rats	↑ Muscle protein	↑ Autophagy signaling ↓ MuRF1, MAFbx	[44]
PYP1-5	/	*Pyropia yezoensis*	Dexamethasone	C2C12 cells	↑ Myotube size	↓ MuRF1, MAFbx	[45]
N-myristoylated Cblin	/	*/*	Dexamethasone	C2C12 cells	↑ Cell diameter	↓ MuRF1, MAFbx	[46]
				C57BL/6 mice	↑ Muscle mass ↑ Muscle fiber cross-sectional areas	↓ MuRF1, MAFbx	

Abbreviation: MAFbx, Muscle atrophy F-box; MAPK, Mitogen-activated protein kinase; mTOR, Mammalian target of rapamycin; MuRF1, Muscle RING-finger protein-1; Myf5, Recombinant myogenic factor 5; MyoD, Myogenic differentiation antigen; SD, Sprague Dawley; ↑, Increase or promote; ↓, Decrease or inhibit.

### 3.2. Minerals

It is well known that minerals play an important role in muscle metabolism and muscle function (Table 2). Calcium and magnesium deficiency induces muscle atrophy in Wistar rats [49]. The fourth Korea National Health and Nutrition Examination Survey data showed that daily calcium intake was significantly lower (2.7%) in subjects with sarcopenia than in those without (6.3%) and there is a strong inverse association between daily calcium intake and sarcopenia in non-obese, older Korean adults [50]. The Maastricht sarcopenia study, which focuses on the differences in nutrient intake and biochemical nutrient status between sarcopenic and nonsarcopenic older adults, suggests that sarcopenic older adults had a 10–18% lower intake of certain nutrients such as magnesium [51]. A randomized controlled trial showed that magnesium supplementation for 12 weeks in healthy elderly women may improve or delay the age-related decline in muscle physical performance and strength [52].

Selenium is an essential trace element for human health and its deficiency is associated with muscle healthy. Chariot and Bignani reported that selenium contributes to the prevention and treatment of muscle disorders, muscle pain, tenderness, muscle fiber atrophy, and physical performance for human in selenium-deficient region [53]. In aged C57BL/6 mice, oral supplementation of selenium significantly augments muscle strength [54]. Besides, supplementation with a combination of fish oil and selenium increase soleus and gastrocnemius muscle mass and reduces IL-6, TNF-*α*, and myostatin levels in gastrocnemius of tumor-bearing C57BL/6 mice [55].

### 3.3. Vitamins

Vitamins play an important role in the prevention of muscular atrophy by inhibiting oxidative stress and related genes (Table 3). A lot of evidence indicated that disturbed redox signaling, due to increased production of ROS and decreased antioxidant capacity, is an important regulator of signaling pathways that control both proteolysis and protein synthesis in skeletal muscle. Vitamin C, a water-soluble vitamin, has the potential for treatment or prevention the treatment of muscle atrophy in mice. Vitamin C deficiency causes muscle atrophy in mice, which is associated with high expression of muscle atrophy gene of MAFbx and MuRF1 and overproduction of ROS. The muscle atrophy was restored by 12 weeks of vitamin C supplementation [56]. In male Wistar rats, oral vitamin C administration can attenuate overload-induced skeletal muscle hypertrophy [57]. Dietary vitamin C supplementation is useful for reducing age-related muscle loss in in middle- and older-aged men and women [58].

Vitamin D deficiency may contribute to the development of muscle atrophy and the possible mechanism is related to the increased expression of MAFbx and protein degradation [59]. Oral administration of vitamin D protects against immobilization-induced muscle atrophy in C57BL/6 mice via vitamin D receptor [60]. Moreover, dietary supplement of vitamin D_3_ significant enhances Wistar rat soleus muscle mass in dexamethasone-induced muscle atrophy model [61].

Vitamin E has been widely investigated as antioxidant interventions to protect against immobilization-induced hindlimb muscle atrophy in Wistar rats [62]. A double-blind randomized controlled trial reported that oral vitamin D and E supplement preserves muscle mass and strength and enhances quality of life in sarcopenic older adults [63]. It was thought that the combination of vitamin C and vitamin E can efficaciously eliminate water-soluble and fat-soluble free radicals at the same time, preventing free radicals from causing oxidative damage to the cell membrane [64]. Thus, future study is warranted to investigate whether supplementation with a combination of vitamin C and vitamin E may have a more pronounced effect compared with the separate treatment of vitamin C and vitamin E. *β*-Carotene, a red-colored pigment found in plants and animals, is one of the most abundant provitamin A carotenoid in human diet and tissues. It attenuated muscle atrophy by repressing the expressions of MAFbx, MuRF1, Ubiquitin-specific processing protease 14, and Ubiquitin-specific processing protease 19 in murine C2C12 myotube and mouse soleus muscle [65].

Oral administration of coenzyme Q_10_ (CoQ_10_), a vitamin-like substance, improves mitochondrial biogenesis and function in skeletal muscle of Goto–Kakizaki rats [66]. Mitochondria biogenesis play a critical role in the metabolic and physiological adaptation of skeletal muscle [67]. Similarly, Liu et al. reported that CoQ_10_ administration ameliorates disuse-induced skeletal muscle atrophy via the activation of mitochondrial biogenesis and the amelioration of oxidative stress in SD rats. In addition, the expression of MuRF-1, MAFbx, and Forkhead box O3 (FoxO3) were reduced by the CoQ_10_ [68].

Nicotinamide mononucleotide is another potential nutraceutical to combat muscle atrophy. Gomes et al. reported that intraperitoneal injection of 500 mg/kg/day nicotinamide mononucleotide for 7 days could reverse muscle atrophy in aged C57BL/6J mice by decreasing the expression of MAFbx and MuRF1 [69]. Long-term administration of nicotinamide mononucleotide enhances age-associated C57BL/6N mice locomotor activity and enhances mitochondrial respiratory capacity in skeletal muscle [70].

Therefore, these results suggest that vitamins have the therapeutic potential for inhibiting muscle atrophy and inducing muscle hypertrophy. The main mechanism is associated with the inhibition of oxidative stress, the activation of mitochondrial biogenesis, and the suppression the expression of genes related to muscular atrophy.

**Table 3:** Vitamins in the prevention of the muscle atrophy in vitro/vivo.

Vitamins	Structure	Example Source	Inducer	Model	Effect	Mechanism	Ref
Vitamin C	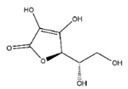	Kiwi fruit, lemons	Senescence marker protein-30-konckout	Mice	↑ Physical performance	↓ MuRF1, MAFbx ↓ Oxidative stress	[56]
Vitamin D	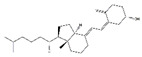	Milk, meat	Immobilization	C57BL/6 mice	↑ Muscle mass	↓ Oxidative stress ↓ MuRF1, MAFbx	[60]
Vitamin E	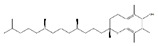	Soybean oil	Immobilization	Wistar rats	/	↓ Oxidative stress	[62]
			Aging	Human	↑ Muscle mass/strength	/	[63]
*β*-Carotene	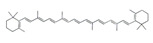	Carrot	Denervation	*ddY* mice	↑ Muscle mass	↓ Oxidative stress ↓ MuRF1, MAFbx, USP14, USP19	[65]
Coenzyme Q_10_	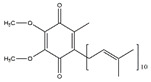	Sardine	Disuse	SD rats	↑ Muscle cross section	↑ Mitochondrial biogenesis ↓ MuRF1, MAFbx, FoxO3	[68]
Nicotinamide mononucleotide	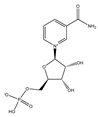	Broccoli, avocado	Aging	C57BL/6N	↑ Physical performance	↑ Mtochondrial oxidative metabolism	[70]

Abbreviation: MAFbx, Muscle atrophy F-box; MuRF1, Muscle RING-finger protein-1; USP 14, Ubiquitin-specific processing protease 14; USP 19, Ubiquitin-specific processing protease 19; FoxO3, Forkhead box O3; ↑, Increase or promote; ↓, Decrease or inhibit.

### 3.4. Fatty Acids

Growing evidence supports the essential role of fatty acid in the regulation of skeletal muscle mass and function (Table 4). For example, docosahexaenoic acid protects mouse C2C12 myoblasts from palmitate-induced atrophy via promoting the expression of peroxisome proliferator-activated receptor *γ* coactiva-tor-1*α* (PGC-1*α*) and attenuating of endoplasmic reticulum stress-associated caspase signaling [71,72]. Woodworth-Hobbs et al. found that docosahexaenoic acid prevents muscle proteolytic induced by palmitate in C2C12 myotubes through the reestablishment of the Akt/FoxO signaling pathway [73]. Similar to docosahexaenoic acid, oleate also prevents palmitate-induced atrophy partly via inhibition of mitochondrial ROS production in C2C12 myotubes [74].

Eicosapentaenoic acid, an omega-3 polyunsaturated fatty acid, has diverse physiological activity including anti-inflammatory and anticachectic actions. Oral administration of eicosapentaenoic acid attenuates arthritis-induced muscle wasting in Wistar rats by decreasing the expression of MAFbx and MuRF1 and increasing the expression of proliferating cell nuclear antigen, MyoD, and myogenin [75].

Dietary supplementation of menhaden fish oil, mainly containing omega-3 unsaturated fatty acids and docosahexaenoic acid, could suppress lipopolysaccharide-induced muscle atrophy in piglets through the modulation of Akt/FoxO, Toll-like receptors and nucleotide-binding oligomerization domain protein signaling pathways [76]. Recently, in another study, dietary menhaden fish oil intake suppresses MuRF1 expression by decreasing TNF-α production in denervation-induced muscle atrophy in C57BL/6J mice [77]. Moreover, a randomized controlled trial suggests that fish oil-derived omega-3 unsaturated fatty acids increase muscle mass and function in healthy older adults [78].

Azelaic acid, a naturally occurring C9 omega-dicarboxylic acid, found in a variety of foods including oatmeal and barley, is a good candidate myogenic molecule that warrants further investigation. Thach et al. reported that administration of azelaic acid stimulates mitochondrial biogenesis, mitochondrial contents, and autophagy in skeletal muscle tissues via activation of olfactory receptor 544 in HFD-induced obese C57BL/6J mice and C2C12 myotubes. The primary mechanism is associated with cyclic adenosine monophosphate-response element binding PGC-1*α*-extracellular signal-regulated kinase-1/2 signaling axis [79]. Arachidonic acid (5,8,11,14-eicosatetraenoic acid C20:4, omega-6) supplementation enhances C2C12 myotubes cell growth and development via cyclooxygenase-2-dependent pathway [80]. Royal jelly is rich in multiple fatty acids, such as trans-10-hydroxy-2-decenoic acid and 10-hydroxydecanoic acid. It attenuates denervation-induced skeletal muscle atrophy in C57BL/6J mice and stimulates myoblast proliferation and differentiation of C2C12 cells [81].

**Table 4:** Fatty acids in the prevention of the muscle atrophy in vitro/vivo.

Fatty Acids	Structure	Example Source	Inducer	Model	Effect	Mechanism	Ref
Docosahexaenoic acid	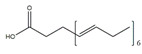	Fish, milk	Palmitate	C2C12 cells	↑ Cell growth	↑ PGC-1*α*, Akt ↓ Endoplasmic reticulum stress	[71,72]
Oleic acid	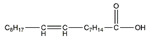	Sesame	Palmitate	C2C12 cells	↑ Myotube size	↓ TNF-*α*, IL6 ↓ Drp1, Fis1 ↓ Myostatin, MAFbx	[74]
Eicosapentaenoic acid	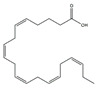	Fish, nut	Arthritis	Wistar rats	↑ Muscle mass	↑ PCNA, MyoD, myogenin ↓ MuRF1, MAFbx	[75]
Arachidonic acid	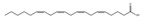	Pine nut	/	C2C12 cells	↑ Cell growth	↑ Cyclooxygenase 2	[80]
Azelaic acid	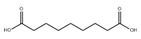	Oatmeal, barley	HFD	C57BL/6J mice	↑ Muscle function	↑ Mitochondrial biogenesis	[79]
			/	C2C12 cells	/	↑ Mitochondrial biogenesis, autophagy	[79]

Abbreviation: Akt, protein kinase B; Drp 1, dynamin-related protein 1; Fis1, Mitochondrial Fission 1 Protein; HFD, high fat diet; IL-6, Interleukin-6; MAFbx, Muscle atrophy F-box; MyoD, Myogenic differentiation antigen; PCNA, Proliferating cell nuclear antigen; PGC-1*α*, Peroxisome proliferator-activated receptor *γ* coactiva-tor-1*α*; TNF-*α*, Tumor necrosis factor-*α*; ↑, Increase or promote; ↓, Decrease or inhibit.

## 4. Phytochemicals

Phytochemicals, powerful nutrient-like substances found in fruits, vegetables, and grains, have shown potential muscle-specific effect in several studies. The results of those studies are reported later separately for different phytochemicals, mainly including polyphenols, flavonoids, polysaccharides, alkaloids, and triterpenoids. Table 2 presents selected articles which highlight the role of phytochemicals in the prevention and treatment of muscle atrophy in various models.

### 4.1. Polyphenols

Polyphenols, a large family of naturally occurring organic compounds characterized by multiples of phenol units, contribute to the prevention of muscle atrophy [82]. Dietary polyphenols are widely distributed in numerous foods including vegetables, fruits, and whole grains [83]. Here, we will summarize the existing scientific evidence gleaned from in vitro and in vivo studies that support the beneficial impacts of dietary polyphenols on the muscle atrophy (Table 5).

Curcumin (1,7-bis-(4-hydroxy-3-methoxyphenyl)-hepta-1,6-diene-3,5-dione), a lipophilic polyphenol and bioactive ingredient of turmeric, alleviates muscle atrophy and promotes muscle health under different conditions. For example, in streptozotocin-induced diabetes C57BL/6J mice dietary administration of curcumin ameliorates muscle atrophy by inhibiting the expressions of MAFbx and MuRF1 [84]. At high altitudes, muscle atrophy caused by chronic hypobaric hypoxia results in decreased physical performance. Curcumin increases the number of muscle fibers and decreases muscle fibers proteolysis in SD rats under hypobaric hypoxia, leading to enhanced muscle mass and improved physical performance [85].

Resveratrol (3, 5, 4′-trihydroxystilbene), a natural antioxidant found mainly in grape skin, and especially red wine, has been shown to inhibit protein degradation and attenuate skeletal muscle fiber atrophy in the different disease model, including cancer, diabetes, and denervation [86,87]. Oral resveratrol treatment alleviates muscle atrophy induced by C26 adenocarcinoma through the inhibition of NF-κB in CD2F1 mice [87]. Resveratrol improves muscle atrophy and enhances grip strength and running distance in streptozotocin-induced diabetic mice, which is associated with a suppression of MuRF-1 and cleaved caspase-3. Besides, it also inhibits mitophagy and increases mitochondrial biogenesis in skeletal muscle of diabetic mice [86]. Moreover, Asami et al. found that dietary resveratrol attenuates muscle atrophy induced by denervation via the downregulation of MAFbx and p62 in institute of cancer research (ICR) mice [88]. Given the reported anti-muscle atrophy effect of resveratrol and the fact that resveratrol acts as a free radical scavenger, it is likely that resveratrol may improve muscle atrophy by removing free radicals in the muscle and reducing muscle oxidative stress; nevertheless, future confirmatory studies are needed.

Astaxanthin, a xanthophyll carotenoid, is a natural antioxidant for oxidative damage. Dietary astaxanthin intake attenuates muscle atrophy caused by immobilization in Wistar mice rats by inhibiting oxidative stress and proteolysis via three major proteolytic pathways, including lysosomal proteases, calpains, and the ubiquitin–proteasome system [102]. Recently, Sun et al. demonstrated that astaxanthin prevents muscle atrophy induced by tail suspension in C57BL/6J mice through protecting the functional stability of mitochondria and alleviating mitochondrial oxidative stress and mitochondria-mediated apoptosis [89].

Tea polyphenols, mainly including epigallocatechin-3-gallate, epigallocatechin, epicatechin-3-gallate, epicatechin, gallocatechins, and gallocatechin gallate, have physiological activities such as antioxidant and anti-inflammation, and also have a positive effect in the prevention and treatment of muscle atrophy [103]. Intraperitoneally administration of epicatechin gallate to C57BL/6 mice stimulates muscle satellite cell activation and differentiation, which is necessary for muscle regeneration. Epicatechin gallate also promotes myogenic differentiation in C2C12 cells [90]. Dietary supplementation of epicatechin prevents muscle loss and regulates the quantity and quality of muscle by increasing the expression of MyoD, superoxide dismutase, and catalase in the biceps femoris muscle and decreasing the expression of FoxO3a, myostatin, and MuRF1 in the soleus muscle of ICR mice [91]. In addition, dietary supplementation of epigallocatechin-3-gallate increases the gastrocnemius muscle mass and the cross-sectional area of muscle fibers in aged SD rats by downregulating MuRF1, MAFbx, and myostatin and increasing IGF-1 mRNA expression [92].

Pomegranate is rich in a variety of phenolic compounds and its extract prevents muscle mass loss induced by TNF-*α* injection in C57BL/6 mice. The mechanism is associated with the inhibition of oxidative stress, NF-κB, and the ubiquitin-proteasome system and the activation of Akt/mTOR signaling [93]. Recently, urolithin A and urolithin B, two novel natural compounds derived from pomegranates, were identified to have preventive effects against muscle trophy. In *C. elegans*, urolithin A, as an autophagy inducer, extends lifespan and improves muscle heath [95]. Mitochondrial autophagy in aging skeletal muscle plays an important role in promoting muscle health. In both in vitro (C2C12 myoblasts and Mode-K intestinal cells) and in vivo (aging and HFD-fed C57BL/6J mice) models, urolithin A induces mitophagy and improves grip strength and overnight running distance [95]. Moreover, oral administration of urolithin A improves mitochondrial function and promotes muscle health in the elderly [94]. Urolithin B increases C2C12 myotubes protein synthesis by activating mTORC1 signaling. In vivo, urolithin B reduces muscle weight loss induced by denervation and inhibit protein degradation by downregulating the mRNA expression of atrophy-related genes, such as FoxO1, FoxO3a, MAFbx and MuRF1 [96].

Glabridin, a major bioactivity compound in licorice derived from the dried roots and rhizomes of *Glycyrrhiza* genus plants and used in both foods and traditional herbal medicine, inhibits dexamethasone-induced protein degradation via downregulating the expression of MuRF1 in C2C12 myotubes [97]. Oral administration of licorice flavonoid oil significantly enhances femoral muscle mass in KK-A^y^ mice and decreases the expression of MuRF1 and MAFbx, which is related to the activation of mTOR, p70S6K, and FoxO3a [104].

Flavan 3-ol fraction, derived from cocoa, enhances muscle atrophy induced by hindlimb suspension in C57BL/6J mice [98]. Oligonol alleviates palmitate-induced senescent phenotype in C2C12 myotubes and suppresses the expression of MAFbx and MuRF1. Moreover, dietary oligonol supplementation reduces the expression of MAFbx and MuRF1 through upregulating sirtuin-1 and FoxO3a in diabetic *db/db* mice [99]. Magnolol attenuates skeletal muscle atrophy in bladder cancer-bearing BALB/c mice undergoing chemotherapy via suppression of FoxO3a and induction of IGF-1 and subsequent downregulation of MuRF-1 and MAFbx [100]. Corylifol A, a phenolic compound from *Psoralea corylifolia* L., enhances myogenesis in C2C12 cells via the activation of p38 MAPKs. It also inhibits dexamethasone-induced muscle atrophy in C2C12 cells, which is related to the inhibition of catabolic pathways and the activation of the anabolic pathway [101].

In addition to above mentioned polyphenols, some extracts rich in polyphenols also with good anti-atrophic activity. For example, chestnut flours extracts, characterized by the composition and quantity of various nutrients and specific bioactive components, such as tocopherols, polyphenols, and sphingolipids, inhibit the expression of MAFbx in C2C12 myotubes muscle atrophy induced by dexamethasone [105]. Red bean extract, abundant in phenolic compounds, significantly increase the phosphorylation of FoxO3 and the protein synthesis associated with PI3K/Akt signaling, and suppresses MuRF1 and MAFbx mRNA expression related with the decreased mRNA level of TNF-α and IL-6 induced by immobilization in C57BL/6N mice, suggesting that red bean extract exerts anti-inflammatory effects by reducing pro-inflammatory cytokines [106].

Overall, polyphenols may be an effective therapeutic strategy for inhibiting muscle atrophy and improving muscle mass and strength. The primary mechanism is related to the inhibition of oxidative stress, inflammation, and muscle atrophy related genes and the activation of IGF-1 signaling pathway. Studies have reported that treatment with combined antioxidant compounds seems more effective compared with single application. For example, the protective effect of a tomato extract containing lycopene, phytofluene, and phytoene on UV-induced erythema formation was more pronounced compared with lycopene treatment alone in human [107]. This could be due to the fact that the interaction between structurally different antioxidant compound may provide more comprehensive protection against oxidative stress. As most of the polyphenols act as antioxidants, it is therefore necessary to investigate the efficacy of a combination of different polyphenol compounds in preventing skeletal muscle atrophy in the future.

### 4.2. Flavonoids

Flavonoids, a class of benzo-gamma-pyrone derivatives abundant in foods and plants, are characterized by a flavone ring and exert anti-inflammation and antioxidant properties [108]. According to different structure characteristics, flavonoids can be divided into several subgroups, including flavonols, isoflavones, chalcones, flavanones, flavanes, flavones, flavanols, flavanonols, and anthocyanidis. Their potential biological activities are highly dependent on specific structures. For example, the metabolism, bioavailability, and biological activity of flavonoids depend upon the configuration, total number of hydroxyl groups, and substitution of functional groups about their nuclear structure [109]. In this section, we will review selected articles where 11 flavonoids were involved and highlight their effect and mechanism in the regulation of muscle atrophy (Table 6).

Apigenin (4′,5,7-Trihydroxyflavone) is a flavonoid found in edible plants including parsley, celery, and grapefruit. Choi et al. reported that dietary supplementation of apigenin ameliorates the obesity-induced skeletal muscle atrophy and increases the exercise capacity. The positive effect of apigenin is related to the upregulation of mitochondrial content and downregulation of gene expression of MuRF1 and MAFbx in skeletal muscle of C57BL/6 mice [110]. The group also reported that in C2C12 myotubes, apigenin suppresses muscle atrophy and mitochondrial dysfunction induced by palmitic acid [110].

Dietary supplementation with quercetin (3,3′,4′,5,7-pentahydroxyflavone), a natural flavonoid mainly present in apples, buckwheat, onions, and citrus fruits, protects against HFD-induced skeletal muscle inflammation and atrophy in C57BL/6 mice by inhibiting the expression of TNF-*α*, monocyte chemoattractant protein 1, MAFbx, and MuRF1 [123]. Kim et al. reported that quercetin suppresses the expression of atrophic factors including MAFbx and MuRF1 induced by TNF-*α* injection under obese conditions in C57BL/6 mice. Furthermore, quercetin enhances heme oxygenase-1 protein level by the activation of nuclear translocation of nuclear factor erythroid 2-related factor 2 (Nrf2) and the inhibition of NF-κB in myotubes [111]. Taken together, quercetin may be used as an outstanding dietary supplement to protect against obesity-induced skeletal muscle atrophy. It is reported that quercetin could counteract the HFD-induced obesity in C57BL/6J mice [123]. Obesity is well known to contribute to skeletal muscle atrophy or sarcopenia. Therefore, it is interesting to investigate whether the anti-muscle atrophy effects of quercetin is related to its anti-obesity effect.

Ampelopsin, a natural flavonoid from Rattan tea, also called dihydromyricetin, has a variety of physiological activities including anti-atrophy effects. Oral administration of ampelopsin improves the d-galactose-induced muscle atrophy in SD rats owing to the decreased MuRF1 and MAFbx and activated adenosine 5′-monophosphate-activated protein kinase (AMPK) and sirtuin-1 signaling pathways [112]. Moreover, ampelopsin administered for 14 days significantly attenuates dexamethasone-induced muscle atrophy in SD rats by reversing mitochondrial dysfunction, which was partially mediated by the PGC-1*α*/mitochondrial transcription factor A (Tfam) and PGC-1*α*/mitofusin-2 signaling pathway [124].

5,7-Dimethoxyflavone, a major bioactive component in *Kaempferia parviflora*, has the pharmacologic effects on skeletal muscle atrophy. Kim and Hwang reported that oral administration of 5,7-dimethoxyflavone improves the exercise capacity and muscle mass by reducing the mRNA expression of MuRF1 and MAFbx in gastrocnemius muscle of eighteen-month-old C57BL/6J mice. 5,7-Dimethoxyflavone also increases mitochondrial DNA content through upregulating PGC-1*α*, nuclear respiratory factor 1, and Tfam in soleus muscle [113].

Intraperitoneal injections of genistein, a soy isoflavone, ameliorates muscle function and morphology in *mdx* mice through inhibiting oxidative stress and blunting proinflammatory factors, such as NF-κB, TNF-*α*, and MAPKs [114]. Whereafter, Aoyama et al. found that dietary genistein supplementation attenuates denervation-induced muscle atrophy in male C57BL/6J mice [115]. Daily intraperitoneal injections of isoflavin-*β*, a mixture of isoflavones, prevents thyrotoxicosis-induced muscle mass loss in Wistar rats through increasing muscle total antioxidant capacity and decreasing mitochondrial cytochrome oxidase activity [116].

Moreover, there are also many flavonoids that have been shown to prevent and treat muscular atrophy. For instance, oral administration of delphinidin prevents disuse muscle atrophy by inducing the miR-23a expression and nuclear factor of activated T cells 3 and suppressing MuRF1 expression in skeletal muscle of C57BL/6J mice [117,118]. Dietary ingestion of 8-prenylnaringenin prevents denervation-induced muscle atrophy in C57BL/6 mice by stimulating the phosphorylation of protein kinase B and downregulating MAFbx [119]. Shukla et al. provided compelling evidence that silibinin increases the protein content and decreases MuRF1 and MAFbx expression in skeletal muscle of pancreatic cancer NCr-nu/nu mice [120]. Dietary supplementation of α-ionone, a naturally occurring flavoring agent in violets and blackberries, improves the loss of skeletal muscle induced by HFD in C57BL/6N mice and prevents C2C12 myotubes from palmitic acid-induced atrophy via cyclic adenosine monophosphate signaling [121]. Cryptotanshinone, a major lipophilic compound found in the root of *Danshen*, protects against muscle atrophy induced by colon adenocarcinoma CT26 in BALB/c mice and improves myotube atrophy of C2C12 cells, which is associated with the reduced the expression of MuRF1 and MAFbx [122].

### 4.3. Polysaccharides

Polysaccharides, the long chain polymeric carbohydrates composed of monosaccharide units bound together by glycosidic linkages, are mainly derived from various plants, fungal and algal sources [125]. Recently, some bioactive polysaccharides have been shown to play an important role in fighting muscle atrophy (Table 7). For example, *Astragalus* polysaccharide, an important bioactive component of *Astragalus membranaceus Bunge* (*Leguminosae*), inhibits dexamethasone-induced muscle atrophy by increasing phosphorylation of Akt, mTOR, P70S6K, and rpS6, and FoxO3a in C2C12 myoblasts. Besides, *Astragalus* polysaccharides also promote cell proliferation and inhibit cell apoptosis in H_2_O_2_-induced cell damage in C2C12 cells [126].

Oral administration of *Aureobasidium pullulans* polysaccharides ameliorates dexamethasone-induced muscle atrophy in ICR mice. The underlying mechanisms involve the increased expression of genes related to muscle protein synthesis (Akt and PI3K) and the inhibition of genes involved in muscle protein degradation (MAFbx, MuRF1, myostatin, and sirtuin-1) [127].

*Dioscorea nipponica* extracts is abundant in polysaccharides and protects against injury-induced muscle atrophy by suppressing NF-κB expression in C57BL/6 mice. In vitro model of C2C12 cells, *Dioscorea nipponica* extracts inhibits TNF-*α*-induced atrophy and promotes myoblast differentiation [129].

Due to the special growth environment, the polysaccharides extracted from marine sources also have biological activities to prevent muscular atrophy. For example, fucoidan, a kind of fucose-enriched sulphated polysaccharides isolated from brown algae, has recently drawn considerable attention owing to against muscle atrophy. Chen et al. reported that dietary supplement of fucoidan can prevent cancer cachexia-associated muscle atrophy in BALB/c mice during chemotherapy, which may be associated with suppressed muscle wasting-related genes including FoxO3a, MuRF1, and MAFbx and enhanced IGF-1-dependent protein synthesis [128]. Oral administration of fucoidan for 4 weeks improves cross-sectional area of extensor digitorum longus fibers and soleus fibers and enhances the expression of MHC in gastrocnemius muscles muscle in C57BL/6 mice [130].

### 4.4. Alkaloids

Alkaloids, a class of structurally diverse array of natural products, have important biological functions on humans and animal. Nine compounds were collected in the literature: matrine, isoquinoline alkaloids, magnoflorine, canadine, tetrahydropalmatine, tomatidine, conessine, theophylline, and apocynin, which are abundant in various plants and show positive physiological effect on muscle atrophy (Table 8).

Alkaloids play an important role in the prevention of muscular atrophy by promoting myoblast differentiation. For example, isoquinoline alkaloids from *Coptis japonica* stimulate myoblast differentiation by promoting MyoD transcriptional activity. Magnoflorine increases expression of MyoD and MHC and subsequently stimulates myoblast differentiation via the activation of p38 MAPK and Akt signaling in C2C12 myotubes [132]. It also significantly decreases the expression of MuRF1 and MAFbx via activating the Akt/mTOR/FoxO signaling pathway in skeletal muscle of diabetic Wistar rats [133]. Canadine, an important alkaloid of *Corydalis turtschaninovii*, stimulates myoblast differentiation through the activation of p38 MAPK and Akt signaling and protects against C2C12 myotube atrophy induced by conditioned media from CT26 colon carcinoma culture by downregulating the muscle specific-E3 ligases, MAFbx and MuRF1 [134]. Tetrahydropalmatine, another compound isolated from *Corydalis turtschaninovii*, enhances myoblast differentiation through the activation of p38MAPK and MyoD and has the potential as a therapeutic candidate to prevent fibrosis and improve muscle regeneration and repair in C2C12 myotubes [135].

Matrine is an alkaloid isolated from the *Sophora flavescens Ait.s* with various pharmacological activities [141]. Matrine improves skeletal muscle atrophy by inhibiting the expression of MuRF1 and MAFbx, which is related to the activation of Akt/mTOR/FoxO3a signaling pathway in dexamethasone-treated C2C12 myotubes and cachexia mice [131].

Tomatidine, a natural small molecule alkaloid abundant in unripe tomatoes, inhibits age-related skeletal muscle atrophy via activating skeletal muscle mTORC1 signaling in C57BL/6 mice. Tomatidine also stimulates mTORC1 signaling resulting in the accumulation of protein and mitochondria, and ultimately, cell growth in C2C12 myoblasts and human skeletal myotubes. Moreover, tomatidine stimulates skeletal muscle hypertrophy and increases strength and exercise capacity [136]. Besides, it improves muscle function during aging in *C. elegans* by activating the Nrf2/Protein skinhead-1 pathway and upregulating mitophagy and antioxidant cellular defenses [137].

Conessine, a steroidal alkaloid, reduces dexamethasone-induced muscle atrophy by downregulating the expression of MuRF1 and MAFbx in C2C12 myotubes [138]. Theophylline reverses the muscle atrophy induced by 28 weeks of cigarette smoke exposure via suppressing the inflammatory effect, upregulating histone deacetylase 2 expression, and inhibiting the activation of NF-κB and p65 in Kunming mice model [139,142]. TGF-*β*, a classical modulator of skeletal muscle, regulates several processes in skeletal muscle disease including inhibition of myogenesis, diminution of regeneration, and induction of muscle atrophy. Apocynin, isolated and purified from *Picrorhiza kurroa Royle ex Benth*, prevents TGF-*β*-induced muscle atrophy in C2C12 myotubes through inhibiting TGF-*β*/Smad signaling [140]. *Tinospora cordifolia* extract rich in alkaloids compounds supplementation improves muscle atrophy induced by sciatic denervation of Swiss albino mice and increases myogenic differentiation of C2C12 myoblasts by alleviating oxidative stress and inflammation [143].

### 4.5. Triterpenoids

Triterpenoids, an enormous range of bioactive compounds derived from methylpentanedioxylic acid with isoprene unit as the basic structural unit, have diverse physiological activity and significant pharmaceutical and industrial applications [144]. This class of natural products includes triterpenes, steroids, limonoids, quassinoids, and triterpenoidal and steroidal saponins (Table 9). Ursolic acid, a pentacyclic triterpenoid abundant in apples, reduces fasting- and denervation-induced muscle atrophy by inhibiting the mRNA expression of MAFbx and MuRF1 and induces skeletal muscle hypertrophy in SD rats [145]. Meanwhile, ursolic acid and low-intensity treadmill exercise significantly improves mass of tibialis anterior and gastrocnemius muscles and decreases atrophy-related gene expression of MuRF1 and MAFbx in SD rats induced by hind limb immobilization [146]. Apple pomace extract is rich in ursolic acid and also plays an important role in the inhibition of muscle atrophy. Dietary supplement with apple pomace extracts significantly increases skeletal muscle strength and weight, which is related to the upregulation the expression of IGF-1 genes and downregulation of the expression of MAFbx and MuRF1 in hindlimb skeletal muscle of C57BL/6N mice. Similarly, it increases the expression of IGF-1, Akt, mTOR, and S6K1 and reduces the expression of MAFbx and MuRF1 in C2C12 myotubes [147].

Ginseng contains a variety of saponins which have beneficial effects on the prevention and treatment of muscle atrophy. For example, ginsenoside Rg1, a primary active ingredient in *Panax ginseng*, increases the viability of mouse C2C12 myoblast cells and inhibits the expression of MAFbx and MuRF1 in the starvation-induced muscle atrophy model. Akt/mTOR/FoxO signaling pathway was involved in this process, suggesting that ginsenoside Rg1 may protect against muscle atrophy by balancing the protein degradation [148]. Like ginsenoside Rg1, ginsenoside Rg3 enhances myotube growth and suppresses the expression of MuRF1, MAFbx in TNF-*α*-induced muscle atrophy of C2C12 myotubes via the activation of Akt/mTOR signaling pathway. Furthermore, ginsenoside Rg3 enhances mitochondrial functions and upregulates the expression of PGC1*α*, Nrf1, and Tfam, and thereby protects TNF-*α*-induced myotube atrophy in C2C12 cells [149]. Additionally, ginsenoside Rb1 and Rb2 increase myotube growth and myogenic differentiation via activating Akt/mTOR signaling pathway [150]. Mountain ginseng, mainly including ginsenoside Rb1 followed by ginsenoside Rb2, attenuates the expression of MuRF1, MAFbx, and FoxO3a and promotes differentiation of myoblasts in dexamethasone-induced L6 cells. In SD rats exposed with dexamethasone, mountain ginseng treatment attenuates skeletal muscle atrophy and inhibits the expression of MuRF1 and MAFbx [151]. Besides, Jiang et al. found that *Panax ginseng* total protein suppresses the dexamethasone-induced muscle atrophy via the AMPK and PI3K/Akt signaling pathways in C2C12 myotubes [152]. Camphene, a cyclic monoterpene found in conifers, reduces starvation-induced oxidative stress and atrophy by regulating ROS generation and lipid metabolism in L6 skeletal muscle cells and SD rats [153].

**Table 9:** Triterpenoids in the prevention of the muscle atrophy in vitro/vivo.

Triterpenoids	Structure	Example Source	Inducer	Model	Effect	Mechanism	Ref
Ursolic acid	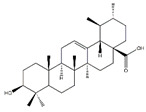	Apples	Hind limb immobilization	SD rats	↑ Muscle mass	↓ MuRF1, MAFbx	[146]
			Denervation	C57BL/6 mice	↑ Muscle mass, muscle hypertrophy	↑ Muscle hypertrophy ↓ MuRF1, MAFbx	[145]
			/	C57BL/6N mice	↑ Muscle weight, muscle strength, exercise capacity	↑ IGF-1 ↓ MuRF1, MAFbx	[147]
			/	C2C12 cells	↑ Myotubes differentiation	↑ IGF-1, Akt, mTOR, S6K1 ↓ MuRF1, MAFbx	[147]
Ginsenoside Rg1	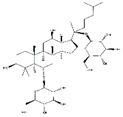	*Panax ginseng*	Starvation	C2C12 cells	↑ Myotubes differentiation	↑ Akt/mTOR/FoxO signaling	[148]
Panax ginseng total protein	/	*Panax ginseng*	Dexamethasone	C2C12 cells	↑ Glucose consumption	↑ AMPK and PI3K/Akt signaling	[152]
Ginsenoside Rg3	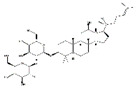	*Panax ginseng*	TNF-*α*	C2C12 cells	↑ Myotubes differentiation	↑ Akt/mTOR signaling ↑ PGC1α, NRF1, Tfam	[149]
Ginsenoside Rb1	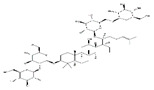	*Panax ginseng*	/	C2C12 cells	↑ Myotubes differentiation	↑ Akt/mTOR signaling	[150]
Ginsenoside Rb2	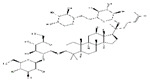						

Abbreviation: Akt, protein kinase B; FoxO, Forkhead box O; IGF-1, Insulin-like growth factor 1; PI3K, Phosphoinositide 3-kinase; MAFbx, Muscle atrophy F-box; mTOR, Mammalian target of rapamycin; MuRF1, Muscle RING-finger protein-1; NRF1, Nuclear respiratory factor 1; PGC1*α*, Peroxisome proliferator-activated receptor *γ* coactiva-tor-1*α*; S6K1, Ribosomal protein S6 kinase; SD, Sprague Dawley; ↑, Increase or promote; ↓, Decrease or inhibit.

### 4.6. Others

*α*-Ketoglutarate rescues corticosterone-induced skeletal muscle atrophy in vitro (C2C12 myotubes) and in vivo (C57BL/6J mice) through inhibiting the expression of MuRF1 and MAFbx [154] (Table 10). Cinnamaldehyde protects H_2_O_2_-induced C2C12 myotubes atrophy by inhibiting the expression of MuRF1 and MAFbx and maintaining the balance of cellular redox [155]. Fucoxanthin, a carotenoid produced by *Undaria pinnatifida* and *Phaeodactylum tricornutum*, increases tibialis anterior muscle and gastrocnemius muscle weight and protects against dexamethasone-induced muscle atrophy through reducing oxidative stress in tibialis anterior muscle in ICR mice [156]. The *Arctium lappa* L. fruit extract rich in arctiin and arctigenin improves cancer-induced muscle atrophy by inhibiting skeletal muscle loss in BALB/c mice. In C2C12 myoblasts and 3T3-L1 cells, it decreases the expression of muscle degradation factors (MAFbx and MuRF1) and increases adipocytes differentiation factors, respectively [157].

Collectively, phytochemicals play an essential role in the prevention and treatment of muscle atrophy, especially in reducing muscle protein degradation and promoting muscle protein synthesis [17,18]. Their primary mechanism includes the enhancement of myoblast differentiation, prevention of muscle protein degradation, the promotion of protein synthesis and myogenesis, anti-inflammation, anti-oxidation, and the downregulation of atrophy-related gene expression. Nevertheless, because the efficacy and molecular mechanisms of most phytochemicals have been examined only in preclinical trials, either in vitro or in vivo, the clinical evidence is not sufficient and is therefore urgently needed. In addition, there is little evidence supporting the structure-function relationship of phytochemicals in skeletal muscle atrophy. The exact relationship between the structure of phytochemicals and their anti-atrophy effect needs to be further investigated.

## 5. Probiotics

Leukemia BALB/c mice, characterized by a loss of fat mass, muscle atrophy, anorexia, and inflammation, has severe dysbiosis of *Lactobacillus*. Whereafter, oral supplementation of *Lactobacillus* (*L. reuteri 100-23* and *L. gasseri 311476*) significantly reduced the expression of atrophy markers (MAFbx and MuRF1) and inflammatory cytokines (IL-6, monocyte chemoattractant protein-1, Interleukin-4, and granulocyte colony-stimulating factor) [158]. In ICR mice, oral administration of *Lactobacillus plantarum* TWK10 for six weeks increases muscle mass and improves exercise performance [159]. Those results suggest that *Lactobacillus* could protect muscle against atrophy and its mechanism is related to decreased systemic inflammation and increased muscle mass.

Inflammatory bowel disease and Crohn’s disease patients suffer from intestinal injury and inflammation-induced muscle atrophy. Oral supplementation *Escherichia coli* significantly decreases the expression of MAFbx and MuRF1 in *B. thailandensis*, *S. Typhimurium* or dextran sulfate sodium-induced muscle atrophy via the activation of the IGF-1/PI3K/Akt signaling pathway in skeletal muscle tissues [160].

Oral administration of *Lactobacillus casei* LC122 and *Bifidobacterium ongum* BL986 for 12 weeks enhances muscle strength and function in C57BL/6 mice [161]. In conclusion, probiotics might potentially constitute a future therapeutic target in the modulation of muscle wasting.

Compared with the above-mentioned dietary nutrients and phytochemicals including polyphenols, flavonoids, polysaccharides, alkaloids, and triterpenoids, knowledge regarding the mechanisms of probiotics is unclear and lacking, while probiotics exert beneficial effect on skeletal muscle atrophy. In this regard, it will be of great interest to delineate the underlying anti-atrophy mechanisms of probiotics in future studies.

Fermented foods, particularly dairy foods, are commonly used as probiotic carriers. For example, the yogurt or fermented milk contains different combinations of probiotics including *Lactobacillus bulgaricus*, *Lactobacillus delbrueckii*, and *Bifidobacterium bifidum* [162]. Iwasa et al. reported that *Lactobacillus helveticus*–fermented milk prevents acute exercise-induced muscle damage [163]. Dietary intake of probiotic kimchi, a representative fermented product containing beneficial bacteria, such as *Leuconostoc mesenteroides* CJ LM119 and *Lactobacillus plantarum* CJ LP133, promotes the amelioration of cachexia-induced muscle atrophy [164]. In addition, prebiotics is known to alter the composition and/or activity of the intestinal microbiota and bring possible benefits to the individual’s muscle health. For example, a randomized controlled double-blind study demonstrated that 13 weeks intake of inulin and fructooligosaccharides complex increases the grip strength in elderly people over 65 years old [165].

Besides, the gut microbiota plays an important role in maintaining host physiology homeostasis by regulating multiple processes, including nutrient absorption, inflammation, oxidative stress, immune function, and anabolic balance. Lahiri et al. found that the expression of MAFbx and MuRF-1 is upregulating in germ-free mice compared to pathogen-free mice. However, when transplanting the gut microbiota from pathogen-free mice into germ-free mice, the skeletal muscle atrophy markers are significantly reduced, and the skeletal muscle mass and oxidative metabolic capacity are improved in C57BL/6J male mice [166], suggesting that gut microbiota may serve an essential role in preventing muscle atrophy. Gut microbiota and their metabolites (e.g., short-chain fatty acids) are likely to engage multiple converging pathways to regulate host muscle growth and function [166]. Considering the reported anti-muscle atrophy effect of some nutraceuticals, such as resveratrol and epicatechin, and the fact that they could modulate the gut microbiome composition and increase circulating levels of short-chain fatty acids [167,168], it is likely that they may improve muscle atrophy by regulating the gut microbiota and their metabolites; nevertheless, future confirmatory studies are needed.

## 6. Conclusions and Perspective

The nutraceuticals reported in this review have been shown to have predominantly preventive effects on muscle atrophy via the attenuation of muscle protein degradation and the promotion of protein synthesis and myogenesis. The mechanisms are associated with the inhibition of inflammation, oxidative stress, glucocorticoid, myostatin, and muscle atrophy-related genes and the activation of IGF-1 signaling pathway. Nutraceuticals with a relevant role in the prevention of muscle protein loss and maintenance of skeletal muscle mass open an important implication in designing appropriate nutritional therapeutic approaches for skeletal muscle atrophy and other muscle health problem.

Although, over the past few decades, the cellular and molecular mechanisms of muscle atrophy have been studied extensively and the beneficial effects of certain nutraceuticals on muscle atrophy in vitro or in vivo have been demonstrated unequivocally, we remain far from having a marketed nutraceutical compounds for muscle atrophy treatment. Future research can focus on the following points: (a) To examine anti-muscle atrophy action and dose of these dietary components in clinical trials as the potentiality of most nutraceuticals has been investigated only in preclinical trials and clinical trials are lacking, and screen the most promising functional ingredient candidates for humans. (b) To study the bioavailability of functional ingredients and their effects on whole body metabolism. (c) To explore more active ingredients with protective ability to muscle atrophy through different experimental methods including 3D cell models, organoid models, and in silico high-throughput screening. (d) To open new frontiers in counteracting imbalances in muscle protein homeostasis by applying new omics science, such as nutrigenomics, which describes the epigenetic mechanisms of action of those nutraceuticals. These will contribute to a more detailed comprehension of muscle atrophy-preventive effects of dietary components, boosting the development of functional ingredients as a tool to counteract muscle atrophy.

## Figures and Tables

**Table 2:** Minerals in the prevention of the muscle atrophy in vitro/vivo.

Minerals	Structure	Example Source	Inducer	Model	Effect	Mechanism	Ref
Magnesium	/	Egg, meat	Aging	Human	↑ Muscle strength	/	[52]
Calcium	/	Egg, meat	Calcium/magnesium deficiency	Human	↑ Muscle mass, strength, and physical performance	/	[50]
Selenium	/	Egg, meat	Selenium deficiency	Human	/	/	[53]
			Aging	C57BL/6 mice	↑ Muscle performance	↑ Ca^2+^ flux, oxidative stress tolerance	[54]
			Tumor	C57BL/6 mice	↓ Inflammation, muscle atrophy	↓ IL-6, TNF-*α*, myostatin	[55]

Abbreviation: IL-6, Interleukin-6; TNF-*α*, Tumor necrosis factor-*α*; ↑, Increase or promote; ↓, Decrease or inhibit.

**Table 5:** Polyphenols in the prevention of the muscle atrophy in vitro/vivo.

Polyphenols	Structure	Example Source	Inducer	Model	Effect	Mechanism	Ref
Curcumin	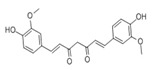	Turmeric	Streptozotocin	C57BL/6J mice	↑ Muscle weight	↓ NF-κB, TNF-*α*, IL-1*β* ↓ MuRF1, MAFbx	[84]
			Chronic hypobaric hypoxia	SD rats	↑ Muscle mass and improved physical performance	↓ Muscle proteolysis	[85]
Resveratrol	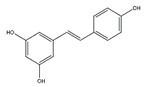	Red wine	Streptozotocin	C57BL/6J mice	↑ Muscle weight, fiber size	↑ Mitochondrial biogenesis ↓ MuRF-1, cleaved caspase-3, mitophagy	[86]
			C26 adenocarcinoma	CD2F1 mice	↑ Muscle mass	↓ NF-κB, MuRF1	[87]
			Denervation	ICR mice	↑ Muscle weight	↓ MAFbx	[88]
Astaxanthin	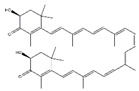	Shrimp, crab	Immobilization	Wistar rats	↑ Muscle mass	↓ Oxidative stress, proteolysis, apoptosis, ROS	[89]
Epicatechin gallate	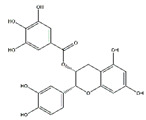	Green tea	Cardiotoxin	C57BL/6 mice	↑ Muscle fiber size	↑ Myf5, MyoD	[90]
Epicatechin	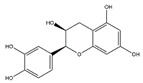	Cocoa, green tea	Aging	ICR mice	↑ Bone mass	↑ MyoD, SOD ↓ MuRF1	[91]
Epigallocatechin-3-gallate	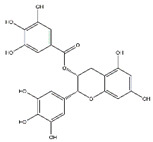	Green tea	Aging	SD rats	↑ Muscle mass, muscle fiber cross-sectional areas	↑ IGF-1, IL-15 ↓ MuRF1, MAFbx	[92]
Pomegranate extract	/	Pomegranate	TNF-*α*	C57BL/6 mice	↑ Muscle mass	↓ Oxidative stress, NF-κB activation, Akt/mTOR signaling	[93]
Urolithin A	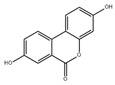	Pomegranate	Aging/	Human	↑ Muscle founction	↑ Mitochondrial founction	[94]
			Aging/HFD	C57BL/6J mice	↑ Muscle founction	↑ Mitochondrial founction	[95]
			/	C2C12 cells	/	↑ Mitochondrial founction	[95]
			/	*C. elegans*	↑ Lifespan, muscle heath	↑ Mitochondrial founction	[95]
Urolithin B	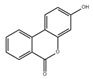	Pomegranate	Denervation	Mice	↑ Muscle weight	↓ FoxO1, FoxO3, MAFbx MuRF1	[96]
			/	C2C12 cells	↑ Protein synthesis	↑ mTORC1 signaling	[96]
Glabridin	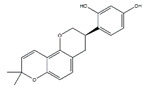	Licorice	Dexamethasone	C2C12 cells	↑ Muscle protein	↓ MuRF1	[97]
Flavan 3-ol	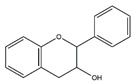	Cocoa	Hindlimb suspension	C57BL/6J mice	↑ Muscle mass	↓ MuRF1	[98]
Oligonol	/	Lychee	/	db/db mice	↑ Muscle fiber size	↑ Sirtuin 1 ↓ MuRF1, MAFbx, FoxO3a	[99]
			Palmitate	C2C12 cells	↑ Myotube differentiation	↓ MuRF1, MAFbx	[99]
Magnolol	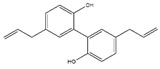	*Magnolia officinalis*	Bladder cancer	BALB/c mice	↑ Muscle protein ↓ inflammation	↑ IGF-1 ↓ MuRF1, MAFbx, FoxO3a	[100]
Corylifol A	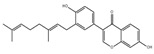	*Psoralea corylifolia* L.	Dexamethasone	C2C12 cells	↑ Mononuclear myotubes	↑ p38 MAPK ↓ MuRF1, MAFbx	[101]

Abbreviation: Akt, protein kinase B; FoxO1, Forkhead box O1; FoxO3, Forkhead box O3; IGF-1, Insulin-like growth factor 1; IL-15, Interleukin-15; MAFbx, Muscle atrophy F-box; MAPK, Mitogen-activated protein kinase; mTOR, Mammalian target of rapamycin; MuRF1, Muscle RING-finger protein-1; Myf5, Recombinant myogenic factor 5; MyoD, Myogenic differentiation antigen; NF-κB, Nuclear factor kappa-B; SD, Sprague Dawley; SOD, Superoxide Dismutase; TNF-*α*, Tumor necrosis factor-*α*; ↑, Increase or promote; ↓, Decrease or inhibit.

**Table 6:** Flavonoids in the prevention of the muscle atrophy in vitro/vivo.

Flavonoids	Structure	Example Source	Inducer	Model	Effect	Mechanism	Ref
Apigenin	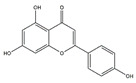	Parsley, celery, and grapefrui	HFD	C57BL/6 mice	↑ Exercise capacity	↓ MuRF1, MAFbx	[110]
			Palmitic acid	C2C12 cells	/	↑ Mitochondrial content	[110]
Quercetin	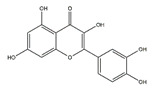	Onions	HFD	C57BL/6J mice	↑ Muscle mass, muscle fiber size	↓ Nrf2, NF-κB, TNF-*α* ↓ MuRF1, MAFbx	[111]
Ampelopsin (dihydromyricetin)	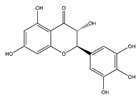	Rattan tea (*Ampelopsis grossedentata*)	D-galactose /Dexamethasone	SD rats	↑ Muscle strength, fiber cross-sectional area	↑ Myotube diameter, mitochondrial content, Tfam, PGC-1*α*	[112]
5,7-Dimethoxyflavone	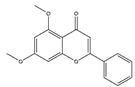	Black ginger (*Kaempferia parviflora*)	Aging	C57BL/6 mice	↑ Muscle mass, strength	↑ Muscle mass, mitochondrial content, Tfam, PGC-1*α* ↓ MuRF1, MAFbx	[113]
Genistein	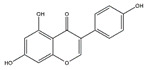	Soybean	/	*mdx* mice	↑ Muscle function and morphology	↑ Muscle function, morphology ↓ NF-κB, TNF-*α*	[114]
			Denervation	C57BL6/J mice	↑ Muscle strength	↓ MuRF1, MAFbx	[115]
Isoflavin-*β*	/	Soybean	Thyrotoxicosis	Wistar rats	↑ Muscle mass	↑ Mitochondrial cytochrome oxidase activity	[116]
Delphinidin	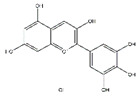	Blueberry	Disuse	C57BL/6J mice	↑ Muscle weight	↑ NFATc3, miR-23a ↓ MuRF1	[117,118]
8-Prenylnaringenin	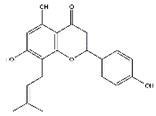	Hop (*Humulus lupulus*)	Denervation	C57BL/6 mice	↑ Muscle weight	↑ Akt, IGF-1	[119]
Silibinin	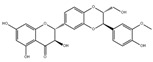	Milk thistle (*Silybum marianum*)	Pancreatic cancer	NCr-nu/nu mice	↑ Muscle strength	↓ MuRF1, MAFbx	[120]
*α*-Ionone	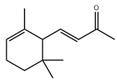	Violets, blackberries, plums	HFD	C57BL/6N	↑ Muscle weight/strength	↑ cAMP ↓ MuRF1, MAFbx	[121]
			Palmitic acid	C2C12 cells	↑ Myotube size	↑ cAMP, MyoD, Myogenin ↓ MuRF1, MAFbx	[121]
Cryptotanshinone	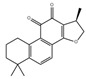	Danshen	CT26 colon carcinoma	BALB/c mice	↑ Muscle mass	↓ MuRF1, MAFbx ↓ STAT3 signaling	[122]
				C2C12 cells	/	↓ MuRF1, MAFbx	[122]

Abbreviation: Akt, Protein kinase B; cAMP, Cyclic adenosine monophosphate; HFD, High fat diet; IGF-1, Insulin-like growth factor 1; MAFbx, Muscle atrophy F-box; MuRF1, Muscle RING-finger protein-1; MyoD, Myogenic differentiation antigen; Nrf2, Nuclear factor erythroid-2 related factor; NF-κB, Nuclear factor kappa-B; PGC-1*α*, Peroxisome proliferator-activated receptor *γ* coactiva-tor-1*α*; STAT3, Signal transducer and activator of transcription 3; TNF-*α*, Tumor necrosis factor-*α*; ↑, Increase or promote; ↓, Decrease or inhibit.

**Table 7:** Polysaccharides in the prevention of the muscle atrophy in vitro/vivo.

Polysaccharides	Structure	Example Source	Inducer	Model	Effect	Mechanism	Ref
*Astragalus* polysaccharide	/	*Astragalus membranaceus Bunge*	Dexamethasone	C2C12 cells	↑ Myotube diameter	↑ Akt, mTOR, p70S6K, rpS6	[126]
Extracellular polysaccharides	/	*Aureobasidium pullulans*	Dexamethasone	ICR mice	↑ Calf thickness, calf muscle strength, gastrocnemius muscle thickness and weight	↑ AkT, PI3K ↓ MAFbx, MuRF1, myostatin, sirtuin 1	[127]
Fucoidan	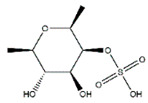	Brown algae	Cancer cachexia, chemotherapy	BALB/c mice	↑ Body weight, muscle weight	↓ Inflammation ↓ FoxO3, MuRF1, MAFbx ↑ IGF-1 signaling	[128]

Abbreviation: Akt, protein kinase B; FoxO3, Forkhead box O3; IGF-1, Insulin-like growth factor 1; MAFbx, Muscle atrophy F-box; mTOR, Mammalian target of rapamycin; MuRF1, Muscle RING-finger protein-1; p70S6K, Ribosomal protein S6 kinase; PI3K, Phosphoinositide 3-kinase; rpS6, Ribosomal protein S6; ↑, Increase or promote; ↓, Decrease or inhibit.

**Table 8:** Alkaloids in the prevention of the muscle atrophy in vitro/vivo.

Alkaloids	Structure	Example Source	Inducer	Model	Effect	Mechanism	Ref
Matrine	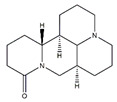	Sophora flavescens	Cachexia	Mice	↑ Muscle fiber size, muscle mass	↓ MuRF1, MAFbx	[131]
			Dexamethasone	C2C12 cells	↓ Apoptosis	↑ Akt/mTOR/p-FoxO3a ↓ MuRF1, MAFbx	[131]
Isoquinoline alkaloids	/	Coptis japonica	/	C2C12 cells	↑ Myoblast differentiation	↑ p38/MAPK, Akt	[132]
Magnoflorine	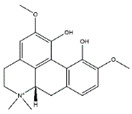	Coptis japonica	CT26 colon carcinoma	C2C12 cells	↑ Myoblast differentiation	↑ p38/MAPK, Akt ↓ MuRF1, MAFbx	[132,133]
			Streptozotocin	Wistar rats	↑ Muscle mass	↓ MAFbx, MuRF-1	[133]
Canadine	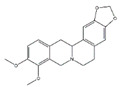	*Corydalis turtschaninovii*	CT26 colon carcinoma	C2C12 cells	↑ Myoblast Differentiation	↑ p38/MAPK, Akt signaling ↓ MAFbx, MuRF1	[134]
Tetrahydropalmatine	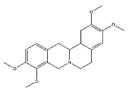	*Corydalis turtschaninovii*	/	C2C12 cells	↑ Myoblast Differentiation	↑ p38/MAPK, Akt signaling	[135]
Tomatidine	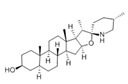	Tomatoes	Aging	C57BL/6 mice	↑ Muscle mass	↑ mTOR signaling	[136]
			/	Human skeletal myotubes, C2C12 cells	↑ Myotube size	↑ mTOR signaling	[136]
			Aging	*C. elegans*	↑ Muscle function	↑ Nrf2 signaling, mitophagy antioxidant	[137]
Conessine	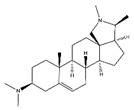	/	H_2_O_2_	C2C12 cells	↑ Myoblast Differentiation	↓ MuRF1, MAFbx	[138]
Theophylline	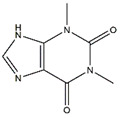	Green tea	Cigarette smoke	Kunming mice	↓ Inflammation	↑ Histone deacetylase 2 ↓ IL-8, TNF-*α*, NF-κB	[139]
			Cigarette smoke extract	C2C12 cells	↓ Inflammation	↑ Histone deacetylase 2 ↓ IL-8, TNF-*α*, NF-κB	[139]
Apocynin	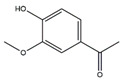	*Picrorhiza kurroa* Royle ex Benth (*Scrophulariaceae*)	TGF-*β*1	C2C12 cells	↑ Myotubes differentiation	↓ TGF-*β*1, ROS	[140]

Abbreviation: Akt, protein kinase B; IL-8, Interleukin-8; MAFbx, Muscle atrophy F-box; MAPK, Mitogen-activated protein kinase; mTOR, Mammalian target of rapamycin; MuRF1, Muscle RING-finger protein-1; NF-κB, Nuclear factor kappa-B; Nrf2, Nuclear factor erythroid-2 related factor; ROS, Reactive oxygen species; TGF-*β*, Transforming growth factor-*β*; TNF-*α*, Tumor necrosis factor-*α*; ↑, Increase or promote; ↓, Decrease or inhibit.

**Table 10:** Other phytochemicals in the prevention of the muscle atrophy in vitro/vivo.

Others	Structure	Example Source	Inducer	Model	Effect	Mechanism	Ref
*α*-Ketoglutarate	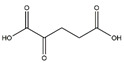	/	Corticosterone	C57BL/6J mice	↑ Muscle mass	↓ MuRF1, MAFbx	[154]
				C2C12 cells	↑ Myotube size	↓ MuRF1, MAFbx	[154]
Cinnamaldehyde	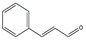	Cinnamon	H_2_O_2_	C2C12 cells	↑ Myotube diameter	↓ MuRF1, MAFbx ↓ Oxidative stress	[155]
Fucoxanthin	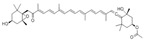	Undaria pinnatifida	Dexamethasone	ICR mice	↑ Muscle mass	↓ Oxidative stress	[156]

Abbreviation: H_2_O_2_, Hydrogen peroxide; MAFbx, Muscle atrophy F-box; MuRF1, Muscle RING-finger protein-1; ↑, Increase or promote; ↓, Decrease or inhibit.

## Data Availability

Not applicable.
